# Low-frequency Raman Spectroscopy as a diagnostic tool for COVID-19 and other coronaviruses

**DOI:** 10.1098/rsos.231568

**Published:** 2023-12-06

**Authors:** The Royal Society Open Science Editorial Team

**A. Stage 1 Title**: Low-frequency Raman Spectroscopy as a diagnostic tool for COVID-19 and other coronaviruses.

**B. Stage 1 Authors and Affiliations: Jacobi, Leor**; Bar-Ilan University, Ramat Gan, Israel. **Damle, Vinayaka Harshothama**; Bar-Ilan University, Ramat Gan, Israel. **Rajeswaran, Bharathi**; Bar-Ilan University, Ramat Gan, Israel. **Tischler, Yaakov R.**; Bar-Ilan University, Ramat Gan, Israel.

**C. Stage 1 Abstract**: Amidst the current worldwide COVID-19 pandemic, improved diagnostic tools are of vital importance. Low-frequency Raman (LFR) Spectroscopy provides a robust theoretical scientific basis for eventual development of a non-invasive Diagnostic Tool for specimens or patients directly. This preliminary research will begin mapping the nanostructure of COVID-19 via LFR and regular Raman Spectroscopy, in comparison with different Coronaviruses and other viral materials, helping to lay a groundwork for future research. In addition to its distinct nanostructure, effects on it by thermal fluctuations or decomposition, decay under laser excitation, and interference from buffers with be examined.

**D. Date of Stage 1 in-principle acceptance:** 8 April 2020.

**E. Date of withdrawal:** 6 April 2021.

**F. Reason for the withdrawal:** Attempts to measure or identify viral material were not successful.

**G. URL to the approved Stage 1 manuscript:** https://osf.io/y54h3.

